# On the inversion-indel distance

**DOI:** 10.1186/1471-2105-14-S15-S3

**Published:** 2013-10-15

**Authors:** Eyla Willing, Simone Zaccaria, Marília DV Braga, Jens Stoye

**Affiliations:** 1 Faculty of Technology, Bielefeld University, Bielefeld, Germany; 2 Institute for Bioinformatics, Center for Biotechnology, Bielefeld University, Bielefeld, Germany; 3 Dip. Informatica Sistemistica e Comunicazione (DISCo), Univ. Milano-Bicocca, Milan, Italy; 4 Inmetro - Instituto Nacional de Metrologia, Qualidade e Tecnologia, Duque de Caxias, Brazil

## Abstract

**Background:**

The inversion distance, that is the distance between two unichromosomal genomes with the same content allowing only inversions of DNA segments, can be computed thanks to a pioneering approach of Hannenhalli and Pevzner in 1995. In 2000, El-Mabrouk extended the inversion model to allow the comparison of unichromosomal genomes with unequal contents, thus insertions and deletions of DNA segments besides inversions. However, an exact algorithm was presented only for the case in which we have insertions alone and no deletion (or *vice versa*), while a heuristic was provided for the symmetric case, that allows both insertions and deletions and is called the inversion-indel distance. In 2005, Yancopoulos, Attie and Friedberg started a new branch of research by introducing the generic double cut and join (DCJ) operation, that can represent several genome rearrangements (including inversions). Among others, the DCJ model gave rise to two important results. First, it has been shown that the inversion distance can be computed in a simpler way with the help of the DCJ operation. Second, the DCJ operation originated the DCJ-indel distance, that allows the comparison of genomes with unequal contents, considering DCJ, insertions and deletions, and can be computed in linear time.

**Results:**

In the present work we put these two results together to solve an open problem, showing that, when the graph that represents the relation between the two compared genomes has no *bad components*, the inversion-indel distance is equal to the DCJ-indel distance. We also give a lower and an upper bound for the inversion-indel distance in the presence of bad components.

## Background

The inversion distance problem in genome comparison searches for the minimum number of signed inversions (reversals) to transform one unichromosomal genome, represented as a signed permutation, into another one with the same gene content and without duplications. The inversion sorting problem requests a sequence of inversions that achieve this minimum number. Hannenhalli and Pevzner (1995) gave the first algorithm for calculating the inversion distance and solving the inversion sorting problem in polynomial time for two linear genomes [1]. Soon after (1997), it was shown that a similar result holds for circular genomes [2]. El-Mabrouk (2000) proposed an extension to include insertions and deletions (indels) to the model [3]. The author introduced an exact algorithm for computing the minimum number of inversion and indel events for the asymmetric case where additional genes are present in only one genome. The symmetric case was treated only heuristically, though.

The double cut and join (DCJ) is an abstract rearrangement operation, introduced by Yancopoulos *et al. *[4] in 2005, which allows to represent most large scale mutation events, such as inversions, translocations, fusions and fissions, which can occur in genomes. If no restriction on the genome structure considering linear and/or circular chromosomes is imposed, using a simple graph data structure, the adjacency graph [5], this leads to considerable algorithmic simplifications. For example, the inversion distance problem can be tackled via the DCJ model in linear time [6].

Yancopoulos and Friedberg [7] introduced insertions and deletions (indels) into the DCJ model but left open the design of an algorithm. This is non-trivial if an indel of consecutive DNA fragments is treated as a single event. In [8] the DCJ distance with indels was considered again, and a linear time algorithm has been proposed. In that paper, the cost of an indel is the same as that of an inversion, but generalizations are possible [9].

In this paper, we combine techniques from [6] and [8] in order to revisit the problem of computing the inversion distance with indels for unichromosomal circular genomes having unequal contents but without duplications. The paper is organized as follows. In the remainder of this section we give definitions and previous results used in this work. We will then use the relational diagram introduced in [10] and prove that, when the graph that represents the relation between the two compared genomes has no *bad components*, the inversion distance with indels equals the DCJ distance with indels, that can be computed in linear time. We then extend the definition of the component tree from [6] in order to give a lower and an upper bound for the inversion distance with indels in the presence of bad components.

### Basic definitions

**Figure 1:**
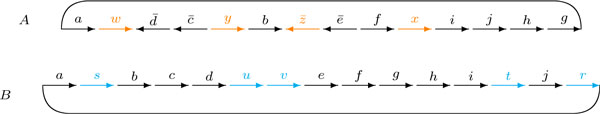
**Graphic representation of the unichromosomal circular genomes *A *and *B*. Each arrow represents a marker and its orientation**. The genome *A*, for example, could be represented by $\left(aw\bar{d}\bar{c}yb\bar{z}ēfxijhg\right)$, or by $\left(cd\bar{w}āḡ\bar{h}\bar{ji}\bar{x}\bar{f}ez\bar{b}ȳ\right)$, or by any circular rotation of these strings.

Each marker in a genome is an oriented DNA fragment. The representation of a marker *g *in a genome *A *can be the symbol *g*, if it is read in direct orientation in *A*, or the symbol ḡ , if it is read in reverse orientation. Let *A *be a unichromosomal circular genome, that is a genome composed of a single circular chromosome. We represent *A *by a string *s*, obtained by the concatenation of all symbols in the chromosome of *A*, read in any of the two directions (we can build *s *starting at any marker). An example is given in Figure 1.

#### Common and unique markers

In this work, duplicated markers are not allowed. Given two unichromosomal circular genomes *A *and *B*, possibly with unequal contents, let G , A  and B  be three disjoint sets, such that G  is the set of *common markers *which occur once in *A *and once in *B*, A  is the set of markers which occur only in *A*, and B  is the set of markers which occur only in *B*. The markers in sets A  and B  are also called *unique markers*. For $A=\left(aw\bar{d}\bar{c}yb\bar{z}ēfxijhg\right)$ and B=(asbcduvefghitjr), we have $G=\left\{a,b,c,d,e,f,g,h,i,j\right\}$, $A=\left\{w,\;x,\;y,\;z\right\}$ and B={r,s,t,u,v}.

#### Indels

In order to sort genomes with unequal contents, we need to consider *insertions *and *deletions *of blocks of contiguous markers [3,8]. We refer to insertions and deletions collectively as *indels*. Indels have two restrictions: (i) markers of G  cannot be deleted; and (ii) an insertion cannot produce duplicated markers [8]. We illustrate an indel with the following example: the deletion of markers *uv *from genome *B *= (*asbcduvefghitjr*) results in *B*' = (*asbcdefghitjr*).

Observe that, if |G|≤1, the problem of sorting *A *into *B *becomes trivial: we simply delete at once the unique content of the chromosome of *A *and insert at once, in the proper orientation, the unique content of the chromosome of *B*. Due to this fact, we assume in this work that |G|≥2.

#### Rearrangements modeled by DCJ

A *double cut and join *(DCJ) [4] is the operation that cuts a genome at two different positions, creating four open ends, and joins these open ends in a different way. Consider, for example, a DCJ applied to genome $A=\left(aw\bar{d}\bar{c}yb\bar{z}ēfxijhg\right)$, that cuts before and after *yb*, creating the segments $∙\bar{z}ēfxijhgaw\bar{d}\bar{c}∙$ and •yb•, where the symbol • represents the open ends. If we then join the first with the third and the second with the fourth open end, we obtain $A^{\prime }=\left(aw\bar{d}\bar{c}\bar{b}ȳ\bar{z}ēfxijhg\right)$. This DCJ corresponds to the inversion of contiguous markers *yb*. The alternative would be to join the first with the second and the third with the fourth open end, giving two circular chromosomes, representing an excision. Its inverse is called an integration, completing the set of DCJ operations for circular genomes [5].

## Methods

In order to find a parsimonious sequence of rearrangements (and indels) sorting one unichromosomal circular genome into the other, it is convenient to find some data structure to represent the relation between the organization of two genomes. This task can be accomplished with the help of the *relational diagram*, proposed in [10]. (Similarly to [11], we adopt here the term *diagram*, as not only the abstract graph structure, but also the linear representation of its nodes along the chromosome is used, as we will describe.) This diagram is a specific view of the *master graph *[12] and unifies in a single structure the *breakpoint diagram*, proposed in [13] to analyze the inversion distance [1] and also used for the inversion-indel distance [3], and the *adjacency graph*, proposed in [5] to analyze the DCJ distance, and then used for the DCJ-indel distance [8].

### The relational diagram

Given two unichromosomal circular genomes *A *and *B*, their *relational diagram*, denoted by *R*(*A, B*), shows the elements of genome *A *in an upper horizontal line and the elements of genome *B *in a lower horizontal line. We denote the two extremities of each marker g∈G by *g^t ^*(tail) and *g^h ^*(head). For each extremity of *g *the diagram *R*(*A, B*) has an orange vertex in the upper line and a blue vertex in the lower line. Clearly, each line (that corresponds to the chromosome of one of the two genomes) has 2|G| vertices, and its vertices are distributed following the same order of the corresponding chromosome. Since the chromosomes are circular, we have to choose one marker a∈G from which we start to read the chromosomes in both genomes, s.t. in both lines the leftmost vertex is *a^h ^*and the rightmost is *a^t^*. Then, for each marker g∈G, we connect the orange and the blue vertices that represent *g^t ^*by a dotted edge. Similarly, we connect the orange and the blue vertices that represent *g^h ^*by a dotted edge.

Moreover, for each integer *i *from 1 to |G|, let γ_1 _and γ_2 _be the orange vertices (analogously blue vertices) at positions 2*i - *1 and 2*i *of the corresponding line of the diagram. We connect the orange vertices (analogously blue vertices) γ_1 _and γ_2 _by an *orange edge *(analogously *blue edge*) labeled by *ℓ*, which is the substring composed of the markers of genome *A *(analogously genome *B*) that are between the extremities represented by γ_1 _and γ_2_. Observe that γ_1 _and γ_2 _are G -*adjacent*, that is, they represent extremities of occurrences of markers from G  in genome *A *(analogously *B*), so that in-between only markers from A  (analogously B ) can appear. In other words, the label *ℓ *contains no marker of G . When the label of an orange (or blue) edge is empty, the edge is said to be *clean*, otherwise it is said to be *labeled*. A similar notion was introduced in [3] as *direct*, resp. *indirect *edge.

**Figure 2:**
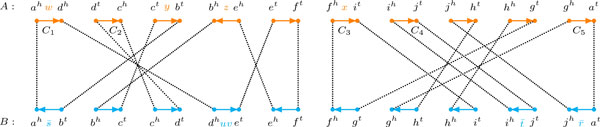
**Example of a relational diagram. For genomes **$A=\left(aw\bar{d}\bar{c}yb\bar{z}ēfxijhg\right)$**and *B *= (*asbcduvefghitjr*) the relational diagram contains five cycles**. Only cycle *C*_2 _is clean, while cycles *C*_1_, *C*_3_, *C*_4 _and *C*_5 _are labeled.

Each vertex is now connected to one dotted edge and either to one orange or to one blue edge, thus the degree of all the vertices is two and the diagram is a simple collection of cycles. Each cycle alternates a pair of orange-dotted with a pair of blue-dotted edges, consequently the length of each cycle is a multiple of 4. By walking through each of these cycles, arbitrarily in one of the two possible directions, we assign an orientation to each colored edge (see Figure 2). The *relative *orientations of the colored edges within one cycle are useful for classifying different types of inversions, as we will see later.

We represent the labels according to the assigned direction instead of taking a simple left-to-right orientation for each edge, in order to avoid any ambiguity. In other words, the orientations of the edges determine the orientations in which the labels are read. Note, however, that an edge $\gamma_{1}\ell \gamma_{2}$ could be equivalently represented as $\gamma_{2}\bar{\ell }\gamma_{1}$. A cycle that contains at least one labeled edge is said to be labeled, otherwise the cycle is said to be clean.

### DCJ sorting and DCJ distance

The cycles of *R*(*A*, *B*) containing only two dotted edges (and one orange and one blue edge) are called 2*-cycles *and are said to be *DCJ-sorted*. Longer cycles are *DCJ-unsorted *and have to be reduced, by applying DCJ operations, to 2-cycles. This procedure is called *DCJ-sorting *of *A *into *B*. A DCJ can be of three types [8]: *split DCJ *when it increases the number of cycles by one; *neutral DCJ *when it does not affect the number of cycles; and *joint DCJ *when it decreases the number of cycles in *R*(*A*, *B*) by one. It has been shown that, given any pair of orange edges (or any pair of blue edges) belonging to the same cycle, a split DCJ can be applied to these edges [14]. (However, depending on the relative orientations of the edges, the number of chromosomes may stay the same, when the DCJ corresponds to an inversion, or increase, when the DCJ corresponds to the excision of a circular chromosome.) Due to this fact, the DCJ distance of *A *and *B*, denoted by *d*_DCJ_(*A*, *B*) and defined as the minimum number of steps required to do a DCJ-sorting of *A *into *B*, is given by the following theorem.

**Theorem 1 **(from [4]). *Given two unichromosomal circular genomes A and B over the same set of markers *G , *we have *$d_{\text{DCJ}}\left(A,B\right)=|G|-c$, *where c is the number of cycles in R*(*A*, *B*).

### Inversion model

**Figure 3:**
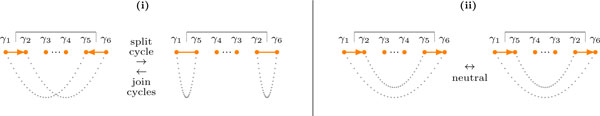
**Effects of an inversion in the diagram (from **[10]**)**. Observe that the inverted segment is inside the horizontal square bracket, that shows γ_2_, γ_3_, ..., γ_4_, γ_5 _at the left side and γ_5_, γ_4_, ..., γ_3_, γ_2 _at the right side of both pictures. **(i) **If the edges are in the same cycle and with opposite orientations, the inversion splits the cycle. Inversely, if the edges are in different cycles, the inversion joins them (independently of the orientations of the original edges, that are omitted). **(ii) **If the edges are in the same cycle with the same orientation, the inversion is neutral and the number of cycles remains unchanged.

In the inversion model, circular excisions and reintegrations are not allowed, and a DCJ can only represent an inversion. In the following, without loss of generality, we will refer to operations applied to orange edges of *R*(*A*, *B*), but a symmetric analysis could be done using blue edges. Differently from a general DCJ operation, an inversion only increases the number of cycles in *R*(*A*, *B*) when it is applied to two orange edges that belong to the same cycle *C *and have opposite orientations according to the arbitrary direction assigned to *C *(see Figure 3) [1].

Two distinct cycles *C *and $C^{\prime }$ are said to be *interleaving *when in the relational diagram there is at least one orange edge of *C *between two orange edges of $C^{\prime }$ and at least one orange edge of $C^{\prime }$ between two orange edges of *C*. An *interleaving path *connecting two distinct cycles *C *and $C^{\prime }$ is defined as the smallest set of cycles *C*_1_, *C*_2_, ..., *C_k _*such that *C*_1 _= *C*, $C_{k}=C^{\prime }$ and *C_i _*and *C_i+1 _*are interleaving for all *i*, 1≤i<k. An *interleaving component *or simply *component *is then a maximal set of cycles  C where each C∈C is connected by an interleaving path to any other $C^{\prime }\in C$.

Components can be of three types. The first type is a 2-cycle, that can never interleave with any other cycle and is then called a *trivial component*. The other two types are components of DCJ-unsorted cycles. Let *C *be a DCJ-unsorted cycle in *R*(*A, B*). If *C *does not have a pair of orange edges with opposite orientations, *C *is called a *bad cycle*. Otherwise the cycle *C *is said to be *good*. A bad cycle *C *cannot be split by any inversion applied to its orange edges. However, if *C *is part of a component  C that contains at least one good cycle, it is always possible to apply one or more inversions that split good cycles of  C, so that *C *becomes good and can then be also sorted with split inversions [1]. Therefore, if a non-trivial component contains at least one good cycle, it is called a *good component*, otherwise it is called a *bad component*.

The relational graph represented in Figure 2 has four components: one good (the cycle *C*_1_), two trivial (the cycles *C*_2 _and *C*_4_) and one bad (composed of the two interleaving bad cycles *C*_3 _and *C*_5_).

When *R*(*A*, *B*) has no bad components, it has been long known that the inversion distance is equal to the DCJ distance:

**Lemma 1 **(adapted from [2,15]). *For two unichromosomal circular genomes A and B, such that R*(*A*, *B*) *has no bad component*, $d_{\text{INV}}\left(A,B\right)=d_{\text{DCJ}}\left(A,B\right)=|G|-c$.

#### Cutting and merging bad components

While the DCJ distance is achieved with split inversions only, bad components require neutral and/or joint inversions to be sorted. Given an inversion *ρ*, we define the DCJ-cost of *ρ*, denoted by ||ρ||, to be respectively 1 or 2 depending on whether *ρ *is a neutral or a joint inversion.

A neutral inversion, applied to any two orange edges of the same bad cycle *C*, turns it into a good cycle [1]. Consequently, if *C *is part of a bad component  C, then  C also becomes a good component. This type of inversion is said to be a *cut *of a bad component. It decreases the number of bad components by one and, since it is a neutral inversion, its DCJ-cost is one.

A joint inversion, applied to two orange edges of two distinct cycles *C*_1 _and *C*_2_, turns them into a single good cycle *C*. If *C*_1 _and *C*_2 _belong to two distinct components $C_{1}$ and $C_{2}$ they are merged into a single good component  C that contains the good cycle *C *[1]. This type of inversion is said to be *a merging *of bad components. It can decrease the number of bad components by at least two, and, since it is a joint inversion, its DCJ-cost is two.

The *inversion distance *between two unichromosomal genomes *A *and *B *with equal content, denoted by *d*_INV_(*A*, *B*), can be then represented by the following equation:

$$d_{\text{INV}}\left(A,B\right)=d_{\text{DCJ}}\left(A,B\right)+\tau_{\text{INV}}\left(A,B\right).$$

The value *τ*_INV_(*A, B*) corresponds to the extra cost for cutting and merging bad components. It can be efficiently computed based on the direct analysis of *R*(*A*, *B*) [1]. In the last section of this paper we will recall an alternative approach [6,16], based on a tree structure that represents the components of *R*(*A*, *B*).

### Runs, indel-potential and the DCJ-indel distance

Now we go back to the general DCJ distance, in which we do not need to take care of bad components. We introduce some definitions and concepts that will help us to integrate indels into the general DCJ model. These concepts are useful to show how to use DCJ operations to minimize the number of indels to be performed. First observe that a set of labels of one genome can be accumulated with DCJs. For example, take the orange edges *c^t^yb^t ^*and $e^{h}\bar{z}b^{h}$ from genome *A *in Figure 2. A DCJ applied to these two edges could result in the new edges *c^t^b^h ^*and $e^{h}\bar{z}ȳb^{t}$, in which the label $\bar{z}ȳ$ results from the accumulation of the labels of the two original edges.

With this notion we can then recall the concept of *run*, introduced in [8]. Given two genomes *A *and *B *and a cycle *C *of *R*(*A*, *B*), a *run *is a maximal subpath of *C*, in which the first and the last edges are labeled and all labeled edges have the same color (belong to the same genome). A run in genome *A *is also called an A -run, and a run in genome *B *is called a B -run. We denote by Λ(*C*) the number of runs in cycle *C*. A cycle has either 0, or 1, or an even number of runs. As an example, note that the cycle *C*_1 _represented in Figure 2 has 4 runs ({*a^h^wd^h^*} and $\left\{e^{h}\bar{z}b^{h},\;b^{h}c^{t},\;c^{t}yb^{t}\right\}$ are A -runs, while $\left\{b^{t}\bar{s}a^{h}\right\}$ and $\left\{d^{h}uve^{t}\right\}$ are B -runs). When we apply split DCJs internal to a single cycle of the relational diagram, we can accumulate an entire run into a single edge [8].

In addition to being accumulated, runs can also be merged by DCJ operations. Consequently, during the optimal DCJ-sorting of a cycle *C*, we can reduce its number of runs. The *indel-potential *of *C*, denoted by *λ*(*C*), is defined in [8] as the minimum number of runs that we can obtain by DCJ-sorting *C *with split DCJ operations. The indel-potential of a cycle depends only on its initial number of runs:

**Proposition 1 **(from [8]). *Given two genomes A and B, the *indel-potential *of a cycle C of R*(*A*, *B*) *is given by *$\lambda \left(C\right)=\left\lceil \frac{\Lambda \left(C\right)+1}{2}\right\rceil$, *if *Λ(C)≥1. *Otherwise, if *Λ(*C*) = 0, *then λ*(*C*) = 0.

Given two unichromosomal circular genomes *A *and *B*, the DCJ distance of *A *and *B *and the indel-potential of the cycles in *R*(*A*, *B*) allow us to easily compute the *DCJ-indel distance*, that is the minimum number of DCJ and indel operations required to sort *A *into *B*, denoted by $d_{\text{DCJ}}^{id}\left(A,B\right)$.

**Theorem 2 **(from [8]). *Given two unichromosomal circular genomes A and B, we have*

$$d_{\text{DCJ}}^{id}\left(A,B\right)=d_{\text{DCJ}}\left(A,B\right)+\underset{C\in R\left(A,B\right)}{\sum }\lambda \left(C\right).$$

## Results

The *inversion-indel distance *between two unichromosomal genomes *A *and *B*, denoted by $d_{\text{INV}}^{id}\left(A,B\right),$ is the number of steps (inversions and indels) required to sort *A *into *B*. It is lower bounded by the DCJ-indel distance and can be represented by the equation

$$d_{\text{INV}}^{id}\left(A,B\right)=d_{\text{DCJ}}^{id}\left(A,B\right)+\tau_{\text{INV}}^{id}\left(A,B\right),$$

in which the value $\tau_{\text{INV}}^{id}\left(A,B\right)$ gives the extra cost to handle bad components of the relational graph.

In this section we present our results, assuming that in *R*(*A*, *B*) the label of each orange edge is composed of at most one marker from A  and the label of each blue edge is composed of at most one marker from B . We first show how to optimally perform indels directly on the original genomes. Then we prove that $\tau_{\text{INV}}^{id}\left(A,B\right)=0$ when *R*(*A*, *B*) has no bad component, and finally we give a lower and an upper bound for $\tau_{\text{INV}}^{id}\left(A,B\right)$ when *R*(*A*, *B*) has bad components.

### Finding optimal integrations

In a DCJ-*indel *sorting scenario there are DCJ operations, insertions of unique markers of B  into *A *and deletions of unique markers of A  from *A*. Although in an arbitrary scenario the order of these operations may vary, from [17] we know that insertions can always be moved ahead of the DCJ operations, s.t. they occur in the first steps, and analogously the deletions can be moved aback to occur after the DCJ operations in the last steps. This separation of insertions, DCJs and deletions within the sorting scenario also appears in [18], where an alternative approach was presented to compute the DCJ-indel distance, based on the concept of *optimal completion*. In this approach, each indel is modeled as a circular chromosome, called *circular singleton*, composed only of the markers that are inserted or deleted by this indel. A completion of genomes *A *and *B *adds *i *new circular singletons to *A *and *k *new circular singletons to *B*, yielding two multichromosomal circular genomes that have the same content G∪A∪B. A completion is optimal when $i+k=\sum_{C\in R\left(A,B\right)}\lambda \left(C\right)$.

Here we show how to build an optimal completion using the relational diagram and the concepts of run and indel-potential. Let *r *be a B -run of a cycle *C *in *R*(*A*, *B*), composed of *m *labels (each label is composed of a single marker, as stated earlier). Then let *s *be the circular singleton obtained from *R*(*A*, *B*) by walking through the path that corresponds to *r *and concatenating its *m *labels. We close the circular chromosome concatenating also the last to the first label. Such a singleton *s *is called *r-singleton*. The addition of the *r*-singleton *s *to genome *A*, yielding genome $A^{\prime }$, produces *m - *1 new clean cycles in the diagram, that is, the number of cycles in *R*(*A*', *B*) is *c' *= *c *+ *m - *1, where *c *is the number of cycles in *R*(*A*, *B*). Since the number of common markers between *A*' and *B *is $|G^{\prime }|\;=\;|G|\;+\;m$, we have *d*_DCJ_(*A*', *B*) = *d*_DCJ_(*A*, *B*) + 1. Furthermore, the cycle *C *in *R*(*A*, *B*) is transformed into a cycle *C*' in *R*(*A*', *B*), containing the same labels of *C *except for the *m *labels of the run *r*.

**Proposition 2**. *If we add the r-singleton of a *B -*run r to genome A yielding genome A*', *the overall indel-potential is achieved, that is, *$\sum_{C^{\prime }\in R\left(A^{\prime },B\right)}\lambda \left(C^{\prime }\right)=\left(\sum_{C\in R\left(A,B\right)}\lambda \left(C\right)\right)-1$*(Analogous for the addition of the r*'*-singleton of an *A -*run r*' *to genome B.)*

*Proof*. Let *C *be the cycle that contains the B -run *r *in *R*(*A*, *B*). We then add the *r*-singleton to genome *A *yielding genome *A*'. If *C *originally had only one or two runs, then it is clear that the sum of the indel-potentials in *R*(*A*', *B*) decreases by one with respect to *R*(*A*, *B*). If *C *originally had four or more runs, two A -runs of *C *are merged into a single run in *R*(*A*', *B*), and this also guarantees that the sum of the indel-potentials decreases by one.    □

For describing the indels in our inversion-indel model, we still need to integrate the singletons so that we obtain a unichromosomal genome. Again, let *r *be a B -run and let *A*' be the genome composed of *A *and the *r*-singleton. We know that *d*_DCJ_(*A*', *B*) = *d*_DCJ_(*A*, *B*) + 1 and, to integrate the singleton, we need to apply exactly one DCJ to two orange (or two blue) edges of a cycle of *R*(*A*', *B*), such that one is part of the chromosome of *A *and the other is part of the *r*-singleton [4,19]. An *optimal integration *is then an integration that preserves the runs of the diagram.

**Proposition 3**. *Any integration of the r-singleton of a *B -*run r into the chromosome of A that creates a new clean cycle in the relational diagram is optimal. (Analogous for the integration of an *A -*run into the chromosome of B.)*

*Proof*. The integration only affects one cycle *C *of the diagram, by splitting it into two cycles. If one of these two cycles is clean, then we know that all runs of *C *remain together in the other cycle, that is, the runs of the diagram are preserved.    □

With the previous results we have a straight recipe for the construction of an *optimal integrated completion *of genomes *A *and *B*. At each step we can decide arbitrarily whether we optimally integrate the *r*-singleton of a B -run to *A*, or the *r*'-singleton of an A -run to *B*, until no more runs exist in the relational diagram. In the end we have two unichromosomal circular genomes A^* ^and B^* ^with the same content.

**Figure 4:**
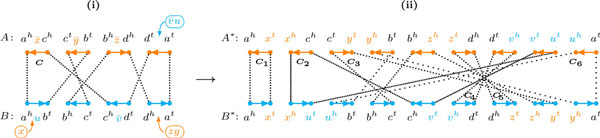
**Optimal integrated completion of two genomes. (i) For genomes **$A=\left(ax\bar{c}yb\bar{z}\bar{d}\right)$**and *B *= (*aubcvd*) we show positions for optimally integrating the singletons in *R*(*A, B*)**. **(ii) **In the resulting genomes $A^{*}=\left(ax\bar{c}yb\bar{z}\bar{d}\bar{v}u\right)$ and B^* ^= (*axubcvdzy*), there are five more common markers between A^* ^and B*, but also five more cycles in *R*(A*, B*).

As an example, let us build one optimal integrated completion for genomes $A=\left(ax\bar{c}yb\bar{z}\bar{d}\right)$ and *B *= (*aubcvd*), whose relational diagram has one cycle *C *with four runs, see Figure 4 (i). We have *λ*(*C*) = 3, thus we need to perform three optimal integrations. We first do an integration of the singleton (*zy*), composed of the labels of an A -run, into the chromosome of genome *B*, creating *B*' = (*aubcvdzy*). After this step, *R*(*A*, *B*') has three cycles, one with two runs. In the second step, we do an integration of the singleton $\left(\bar{v}u\right)$, composed of the labels of the last B -run, into the chromosome of genome *A*, creating $A^{*}=\left(ax\bar{c}yb\bar{z}\bar{d}\bar{v}u\right)$. Now *R*(A*, *B*') has five cycles, one with an A -run. We finally do an integration of the singleton (*x*), composed of the labels of the last A -run, into the chromosome of genome *B*', creating B^* ^= (*axubcvdzy*), yielding *R*(A*, B*) composed of six clean cycles, see Figure 4 (ii). Indeed, *d*_DCJ_(*A*, *B*) = *d*_DCJ_(A*, B*).

### Finding safe integrations - the inversion-indel distance in the absence of bad components

Let *A *and *B *be two unichromosomal circular genomes with unequal contents such that *R*(*A*, *B*) has no bad component. A *safe integration *is an optimal integration in *A *yielding *A*' (respectively in *B *yielding *B*'), such that also *R*(*A*', *B*) (respectively *R*(*A*, *B*')) has no bad component.

**Figure 5:**
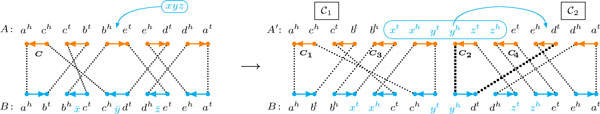
**Optimal but not safe integration. For genomes $A=\left(a\bar{c}bed\right)$ and *B *= (*abxcydze*), an optimal but not safe integration of the singleton (*xyz*) produces *A'***. In *R*(*A*', *B*) we have two clean 2-cycles (*C*_3 _and *C*_4_), one good component $C_{1}=\left\{C_{1}\right\}$ and one bad component $C_{2}=\left\{C_{2}\right\}$. The marker *y *is a link of $C_{1}$ and $C_{2}$ and is adjacent to *d *in genome *B*. This information is used to find an alternative optimal integration for the singleton (*xyz*), as we will show in Figure 6.

In Figure 5 we perform an optimal but not safe integration, producing a bad component in the relational diagram. Even several bad components can be created by an optimal integration, but, fortunately, it is always possible to perform a safe integration, as shown in the following.

Let the *size *of a component  C in *R*(*A*, *B*) be the total number of orange (or blue) edges in the cycles of  C. Furthermore, let $C_{1}$ and $C_{2}$ be two components in *R*(*A*, *B*). If each orange edge of $C_{1}$ is between two orange edges of $C_{2}$, the component $C_{1}$ is said to be *nested *within $C_{2}$. Otherwise, if $C_{1}$ is not nested within *C*_2 _and *C*_2 _is not nested within $C_{1}$, the components $C_{1}$ and $C_{2}$ are said to be *independent*. Two independent components $C_{1}$ and $C_{2}$ are said to be *linked *if the leftmost orange edge of $C_{2}$ appears immediately after the rightmost orange edge of $C_{1}$ in *R*(*A*, *B*). In this case the rightmost orange vertex of $C_{1}$ and the leftmost orange vertex of $C_{2}$ represent extremities of the same marker g∈G. The marker *g *is said to be a *link *of $C_{1}$ and $C_{2}$. A sequence of *k *linked components is called a *chain *of size *k*.

**Figure 6:**
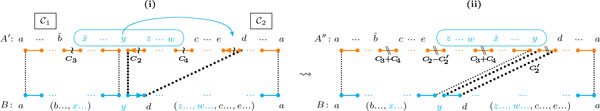
**Our approach to find an alternative to an optimal integration that creates a bad component**. Observe that, from *R*(*A*', *B*) to *R*(*A*", *B*), only the orange edges marked with the symbol ≀ were transformed into the orange edges marked with the symbol \\. All the other edges of the diagram were preserved. While the distinct cycles *C*_3 _and *C*_4 _of *R*(*A*', *B*) are merged into a single cycle in *R*(*A*", *B*), the cycle *C*_2 _of *R*(*A*',*B*) is split into two cycles in *R*(*A*", *B*). The hat on markers *b *and *x *indicates that we make no assumptions about the orientation of theses markers (but we know they have the same orientation in *A*' and *A*"). **(i) **After the first integration we have a good component $C_{1}$ at the left side, and a bad component $C_{2}$ at the right side (at the interval *yz...wc...ed...a *of *A*'). The marker *y *is a link of $C_{1}$ and $C_{2}$ and is adjacent to *d *in genome *B*. **(ii) **If we do the optimal integration inside $C_{2}$, so that *y *is adjacent to *d *in genome *A*", we create the clean 2-cycle $C_{2}^{\prime }.$ There can be a bad component in *R*(*A*", *B*) (at the interval *c...ez...w *of *A*"), but it is strictly smaller than *C*_2_.

Without loss of generality, let all markers in *B *have the same orientation and let *R*(*A*, *B*) have only one component  C, that is good. Assume that an optimal integration of a singleton *s *in *A *yielding *A*' creates, besides one or two trivial components, exactly one good component $C_{1}$ and one bad component $C_{2}$ in *R*(*A*', *B*). If necessary, we can flip genome *A*' so that the markers within $C_{2}$ in *A*' have the same orientation as the markers in *B*. Furthermore, due to the circularity of the genomes, we can rotate the diagram so that *R*(*A*', *B*) is a chain of exactly two linked components $C_{1}$ and $C_{2}$. A link of $C_{1}$ and $C_{2}$ is within the optimal integration. If we then do an alternative optimal integration of *s *in the middle of the bad component $C_{2}$ (see Figure 6), we obtain *A*". In *R*(*A*", *B*) we have either a single bad component smaller than $C_{2},$ or no bad component.

(In general, there can be other components in *R*(*A*', *B*) nested within $C_{1}$ and $C_{2}$, but each one of these is either trivial or has at least one edge within and at least one edge outside the integrated cluster. In any case, since the component in *R*(*A*, *B*) was good, at least one component in *R*(*A*', *B*) has to be good. By extending the approach illustrated in Figure 6 we can show that all components but $C_{2}$ are merged into a single good component and only one bad component, strictly smaller than $C_{2}$, can exist in *R*(*A*", *B*).)

**Proposition 4**. *Let r be a *B -*run in R*(*A, B*). *At least one optimal integration of the r-singleton into the chromosome of A is safe. (Analogous for the integration of an *A -*run in B.)*

*Proof*. Assume that each optimal integration of the *r*-singleton in *A*, yielding *A*', creates at least one bad component in *R*(*A*', *B*). Then, among all possible optimal integrations of *r*, assume that we take one that produces a bad component $C^{\prime }$ of the smallest size. It is always possible to perform another optimal integration of *r*, as described in Figure 6, in the middle of the bad component $C^{\prime },$ transforming *A*' into *A*", so that we create a clean 2-cycle in *R*(*A*", *B*). Either *R*(*A*", *B*) does not have any bad component (then we have a contradiction to the assumption that all optimal integrations create bad components), or it has a bad component $C^{\prime \prime }$ (then $C^{\prime \prime }$ must be strictly smaller than $C^{\prime }$, and we have a contradiction to the assumption that $C^{\prime }$ was a bad component with the smallest size).    □

The results presented above give rise to the following theorem:

**Theorem 3**. *For two unichromosomal circular genomes A and B, such that R*(*A*, *B*) *has no bad component, we have *$d_{\text{INV}}^{id}\left(A,B\right)=d_{\text{DCJ}}^{id}\left(A,B\right)$.

*Proof*. We know that there is at least one safe integration for each run and that by integrating one run per step we perform exactly $\sum_{C\in R\left(A,B\right)}\lambda \left(C\right)$ integrations, yielding genomes A^* ^and B^* ^with the same content, such that *R*(A*, B*) has no bad component. Then we have *d*_DCJ_(*A, B*) = *d*_DCJ_(A*, B*) = *d*_INV_(A*, B*).    □

Since the DCJ-indel distance can be computed in linear time, the same is true for the inversion-indel distance in the absence of bad components.

### Bounds for the inversion-indel distance in the presence of bad components

Now we will give bounds to the extra cost for handling bad components in *R*(*A*, *B*). Without loss of generality, let us assume that, if *R*(*A*, *B*) has at least two components, the first and the last orange edges of *R*(*A*, *B*) belong to two distinct components. Recall that *R*(*A*, *B*) represents the relation between two circular chromosomes, thus its first orange edge comes right after its last orange edge.

Let $C_{1}$, $C_{2}$ and $C_{3}$ be three distinct components in *R*(*A*, *B*) such that if we take the rightmost orange edge of $C_{1}$ and look at the following orange edges one by one, we always find an edge of $C_{3}$, before finding an edge of $C_{2}$. In the same way, if we take the rightmost orange edge of $C_{2}$ and look at the following orange edges one by one, we always find an edge of $C_{3}$, before finding an edge of $C_{1}$. The component $C_{3}$, is then said to *separate *$C_{1}$ and $C_{2}$. (In Figure 2 the good component {*C*_1_} separates the trivial component {*C*_2_} from both the trivial component {*C*_4_} and the bad component {*C*_3_, *C*_5_}. Similarly, {*C*_3_, *C*_5_} separates {*C*_4_} from both {*C*_2_} and {*C*_1_}.) By joining two cycles *C*_1 _and *C*_2_, that belong to two distinct components $C_{1}$ and $C_{2}$, we merge not only the components $C_{1}$ and $C_{2}$, but also all components that separate $C_{1}$ and $C_{2}$, into a single component  C. Even when all merged components are bad, the new component  C is always good [1].

The extra cost for handling bad components can be computed using an approach from [6,16], in which a tree structure is defined representing the linking and nesting relationship of the components of *R*(*A*, *B*).

#### The component tree

The *component tree T *(*A*, *B*) is a rooted tree with two types of nodes, defined as follows [16]:

1. Each component is represented by a *round node*.

2. Each maximal chain is represented by a *square node *whose children are the round nodes that represent the components of this chain.

3. A square node is either the root, or the child of the smallest component in which this chain is nested.

**Figure 7:**
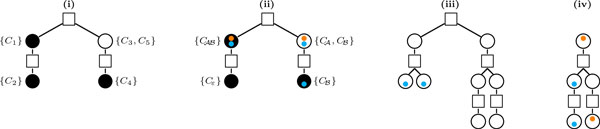
**Examples of component trees. (i) The tree *T *(*A*, *B*) for the relational diagram represented in Figure 2 has one bad (white) and three good (black) nodes, and (ii) the corresponding colored tree *T*_o_(*A, B*)**. Here, the indel-type of each cycle is given. In both cases the trees $T^{\prime }$ and $T_{o}^{\prime }$ are composed of a single bad node. **(iii) **An example of a $T_{o}^{\prime }$ to show that a greedy strategy, of maximizing the merging of leaves with the same colored dot, does not work. If we merge the two leaves with blue dots the cost of the cover is 5. However, if we merge twice a leaf with a blue dot and a leaf with no dot (the longer paths), the cost is 4. **(iv) **Another example of a $T_{o}^{\prime }$ to show that, on the other hand, if we merge the leaves of the longer path we have a cost of 3. But if instead we merge the two nodes with blue dots and the two nodes with orange dots, the cost is 2.

A round node is called a *bad node*, drawn in white, if it represents a bad component. Otherwise it is called a *good node*, drawn in black. (A good node can be a trivial or a good component.) Figure 7 (i) shows an example of *T *(*A*, *B*).

**Reducing *T *to *T'***. Let *T' *be the unrooted tree that corresponds to the smallest subgraph of *T *(*A*, *B*) that contains all bad nodes. Let a *long branch *be a branch in *T' *that contains two or more bad nodes.

**Covering the bad nodes**. A path *P *in *T' *can be short, if *P *contains only one vertex, or long, if *P *contains at least two vertices. A *cover *of *T' *is defined as a set of paths that contain all bad nodes of *T'*. The cost of a cover is given by the sum of the costs of its paths and an *optimal cover *of *T' *is a cover with the minimum cost.

**Computing *τ*_INV _(*A*, *B*)**. For the inversion model, by assigning the cost of one to each short path and the cost of two to each long path, it has been shown in [6,16] that the cost of an optimal cover of *T' *corresponds exactly to the value *τ*_INV_(*A*, *B*) and can be computed as follows:

**Theorem 4 **(from [6,16]). *Let w be the number of leaves of T'. Then*

$$\tau_{\text{INV}}\left(A,B\right)=\left\{\begin{array}{cc} w+1 & if\;w\;is\;odd\;and\;all\;leaves\;are\;on\;long\;branches,\\ w & otherwise. \end{array}\right)$$

#### The costs of cutting and merging bad components in the inversion-indel model

Recall that the DCJ-cost of an inversion *ρ *is denoted by ||ρ|| and corresponds respectively to 1 or 2 depending on whether *ρ *is a neutral or a joint inversion. Furthermore, let *λ*_0 _and *λ*_1 _be, respectively, the sum of the indel-potentials for the components of the relational diagram before and after the inversion *ρ*. We then have Δ*λ*(*ρ*) = *λ*_1 _- *λ*_0 _and we also define the cost of *ρ *to be Δd(ρ)=||ρ||+Δλ(ρ).

Each cut is a neutral inversion *ρ *that has ||ρ||=1. If *ρ *cuts a bad component  C that contains only cycles with at most two runs, it is clear that *ρ *cannot save indels. In this case, Δ*d*(*ρ*) = 1. However, if  C contains a cycle *C *with at least four runs, it is possible to apply *ρ *such that two A -runs and two B -runs are merged. This reduces the number of runs by two, that is, ΔΛ(*ρ*) = -2, hence Δλ(*ρ*) = -1 and Δ*d*(*ρ*) = 0.

Each merging is a joint inversion *ρ *that has ||ρ||=2. The cost of each merging depends on the runs of the affected cycles. A cycle with no run is represented by *C_ε_*. Let $C_{A}$ (respectively $C_{B}$) be a cycle with an A -run (respectively a B -run). Similarly, let $C_{AB}$, be a cycle with two or more runs. In Table 1 we show the costs of the different types of joint inversions.

**Table 1:** Types of joint inversions (C_* _represents a cycle with any number of runs, Δ*d*(*ρ*) = 2 + Δ*λ*(*ρ*)).

sources	resultant	Δλ(*ρ*)	Δ*d*(*ρ*)
*C_ε _*+ C_*_	C_*_	0	2
$C_{A}\;+\;C_{B}$	$C_{AB}$	0	2
$C_{AB}\;+\;C_{AB}$	$C_{AB}$	-2	0
$C_{A}\;+\;C_{A}$	$C_{A}$	-1	1
$C_{B}\;+\;C_{B}$	$C_{B}$	-1	1
$C_{A}\;+\;C_{AB}$	$C_{AB}$	-1	1
$C_{B}\;+\;C_{AB}$	$C_{AB}$	-1	1

#### The colored component tree

All components that have a cycle of type $C_{AB}$ can be merged together into a single (good) component with cost 0, thus we assume that *R*(*A*, *B*) has at most one component  C of this type. Furthermore, if  C is bad, we also assume that it has no cycle with four or more runs. (Otherwise it could be cut with cost 0.)

With these assumptions, we build the component tree *T *(*A*, *B*) as described previously. Then we transform *T *(*A*, *B*) into *T*_o_(*A*, *B*), by adding at most two colored dots to each round node, as follows: we add an orange dot, if at least one cycle of the corresponding component has an A -run; and a blue dot, if at least one cycle of the corresponding component has a B -run. Figure 7 (ii) shows an example of *T*_o_(*A*, *B*).

**Reducing *T*_o _to **$T_{o}^{\prime }$ Let $T_{o}^{\prime }$ be the unrooted tree that corresponds to the smallest subgraph of *T*_o_(*A, B*) that contains all bad nodes. The leaves of $T_{o}^{\prime }$ are bad components. Let *v *be a leaf of $T_{o}^{\prime }$ and let *t *be the subtree of *T*_o_(*A*, *B*) rooted at *v*. In $T_{o}^{\prime }$, the leaf *v *will then have the union of all colored dots from *t*.

**Computing **$\tau_{\text{INV}}^{id}\left(A,\;B\right)$. The cost of a short path here is also one. On the other hand, the cost of a long path is either one, if its endpoints share at least one colored dot, or two otherwise. An optimal cover of $T_{o}^{\prime }$ corresponds to the value of $\tau_{\text{INV}}^{id}\left(A,\;B\right)$. However, the problem of computing this value is very intricate, even when each node has at most one colored dot, as we can see in Figure 7 (iii) and (iv).

Below we give a lower and an upper bound for $\tau_{\text{INV}}^{id}\left(A,\;B\right)$, but finding an exact formula to compute this value is left as an open problem.

**Proposition 5**. *Let *$\tau_{\text{INV}}^{id}\left(A,\;B\right)$*be the cost of an optimal cover of *$T_{o}^{\prime }$. *We then have:*

$$\left\lceil \frac{w}{2}\right\rceil \leq \tau_{\text{INV}}^{id}\left(A,\;B\right)\leq w+1,$$

*where w is the number of leaves in *$T_{o}^{\prime }$.

*Proof*. The lower bound can be obtained when *w *≤ 1 or when all leaves share at least one colored dot (in this case, all paths have cost 1). The upper bound occurs when *w *is odd, all leaves are clean (have no colored dot) and are on long branches (the greatest value of Theorem 4).    □

## Conclusions

In this work we have revisited the inversion-indel distance between two unichromosomal genomes *A *and *B *with unequal contents. We have shown that, when the relational diagram *R*(*A*, *B*) has no bad component, the inversion-indel distance is equal to the DCJ-indel distance of *A *and *B *and can be computed in linear time. We also gave a lower and an upper bound for the extra cost $\tau_{\text{INV}}^{id}\left(A,\;B\right)$ of handling bad components in *R*(*A*, *B*). However, finding an exact formula to compute this value is very intricate and was left as an open problem.

## Competing interests

The authors declare that they have no competing interests.

## Authors' contributions

EW, SZ, MDVB and JS have elaborated the model, proved the results and written the paper.

## Declaration

MDVB is funded by the Brazilian research agency CNPq grant PROMETRO 563087/10-2. The Deutsche Forschungsgemeinschaft and the Open Access Publication Funds of Bielefeld University Library supported the Article Processing Charge.

This article has been published as part of *BMC Bioinformatics *Volume 14 Supplement 15, 2013: Proceedings from the Eleventh Annual Research in Computational Molecular Biology (RECOMB) Satellite Workshop on Comparative Genomics. The full contents of the supplement are available online at http://www.biomedcentral.com/bmcbioinformatics/supplements/14/S15.
